# Molecular Identification, Occurrence, and Risk Factors for Small *Babesia* Species Among American Stafford Terriers in Serbia

**DOI:** 10.3390/vetsci13010026

**Published:** 2025-12-25

**Authors:** Dajana Davitkov, Milica Kovačević Filipović, Dimitrije Glišić, Elmin Tarić, Anja Ilić Bozović, Milena Radaković, Darko Davitkov

**Affiliations:** 1 Faculty of Veterinary Medicine, University of Belgrade, Bulevar Oslobođenja 18, 11000 Belgrade, Serbia; dajana@vet.bg.ac.rs (D.D.); milica@vet.bg.ac.rs (M.K.F.); etar1989@yahoo.com (E.T.); bozovicia@vet.bg.ac.rs (A.I.B.); davitkov@vet.bg.ac.rs (D.D.); 2 Institute of Veterinary Medicine of Serbia, Janisa Janulisa 14, 11000 Belgrade, Serbia; dimitrije.glisic@nivs.rs

**Keywords:** American Staffordshire Terriers, DNA analysis, Serbia, prevalence, vector-borne disease

## Abstract

In Serbia, *Babesia canis* is the predominant cause of canine babesiosis, while *B. gibsoni* and *B. vulpes* have been reported only sporadically. Limited awareness of these small Babesia species often leads to misdiagnosis and inappropriate treatment. International reports indicate that American Staffordshire Terriers (ASTs) and related breeds are particularly affected. This study examined 101 ASTs over a two-year period to determine the occurrence of small *Babesia* species and identify dog- and owner-related risk factors. Collected data included age, sex, body condition score (BCS), clinical signs, presence of scars, history of vector-borne diseases (VBD), living conditions, antiparasitic prophylaxis, and tick exposure. Molecular identification using PCR and Sanger sequencing revealed that 37 dogs were positive for *Babesia* spp.; among them, 36 were infected with *B. gibsoni* and one with *B. vulpes*. Significant risk factors associated with infection included the presence of scars, low BCS, history of VBDs, tick exposure, and living in rural areas. These findings demonstrate a notably high occurrence of *B. gibsoni* and the presence of *B. vulpes* in ASTs in Serbia. The results highlight the need for improved awareness, accurate molecular diagnostics, and targeted preventive and therapeutic measures. Additionally, observed risk factors suggest chronic infection and support transmission pathways linked to dog bites and tick exposure.

## Simple Summary

In Serbia, most canine babesiosis cases are caused by *Babesia canis*, but infections with small *Babesia* species, especially *Babesia gibsoni*, are often overlooked and incorrectly treated. These species require different therapy and can cause long-lasting, chronic infections. In this study, we examined 101 American Staffordshire Terriers (ASTs) over two years. Using molecular methods, we found that 37 dogs were infected: 36 with *B. gibsoni* and one with *B. vulpes*. The main risk factors were the presence of scars (possible bite wounds), low body condition, previous vector-borne diseases, tick exposure, and living in rural areas. These findings show that small *Babesia* species are more common in ASTs than previously thought and highlight the importance of proper diagnosis, tick prevention, and reducing dog-to-dog bite transmission.

## 1. Introduction

Belgrade, Serbia’s capital, has a population of about two million people and an estimated half-million dogs. The city is considered endemic for canine babesiosis [1], a disease caused by several *Babesia* species transmitted through the bites of various tick vectors. The first cases were documented in 1994 [2], but advances in molecular diagnostics have only recently revealed that the majority of acute clinical cases are attributable to *B. canis* infection, with a smaller number linked to *B. gibsoni* [3,4] and *B. vogeli* [5]. Outdoor dogs are considered asymptomatic carriers of *B. canis* and *B. vogeli*, with seroprevalence observed in approximately one-third of tested individuals [6]. Additionally, a smaller subset of asymptomatic dogs has been confirmed to harbour *B. canis* or *B. gibsoni* DNA in their blood samples [6]. Although *B. vulpes* is acknowledged as a highly pathogenic canine pathogen [7], it has not been identified as a causative agent of canine babesiosis in Serbia. Nevertheless, stray dogs, foxes, and jackals in Serbia harbour this parasite [8,9]. Two recent cases of infection with *B. gibsoni* in Serbia have underscored the importance of *B. gibsoni* [4]. These parasites are challenging to detect on blood smears, often exhibit resistance to commonly used antibiotics and antimalarial regimens, and can induce secondary immune-mediated hemolytic anemia [4]. These underscore the growing clinical and epidemiological significance of small *Babesia* species as emerging canine pathogens in Serbia. Globally, *B. gibsoni* is the most frequently identified *Babesia* species in dogs, accounting for approximately 50% of cases [10]. To date, various dog breeds in Asia have been documented as infected [11]. However, in Sri Lanka, Rottweilers are at a higher risk of *B. gibsoni* infection than other breeds [12], while in Japan, the same applies to Tosa dogs [13]. In contrast, most cases reported in North America and Europe involve American Staffordshire Terriers (ASTs) and related breeds [14,15]. The tick vectors for *B. gibsoni* identified in Asia—*Haemaphysalis longicornis* [11] and *H. hystricis* [16]—have not been confirmed in Europe [17]. The proposed, but not yet confirmed, European vector is *Rhipicephalus sanguineus* [17,18]. Meanwhile, the primary candidate vectors for *B. vulpes*—*Ixodes hexagonus* and *I. canisuga* [17,19]—are present in Serbia, where they have been documented in wild red foxes [20]. In the absence of confirmed vectors, alternative transmission routes merit consideration. Transplacental transmission [21], transfusion-related infections, history of travel in *B. gibsoni* endemic areas and non-compliance with aseptic and antiseptic practices in patient care [22] are possible. Additionally, various risk factors indicating *B. gibsoni* infection, dependent on dogs (sex, breed, age) and their owners (ectoparasite control, indoor/outdoor housing), have been studied [12,23,24,25]. The results are inconsistent, indicating that the risk factors, especially across the European continent, have not yet been thoroughly investigated. The popularity of ASTs in Serbia, and Belgrade, has been rising over the last decade. This breed was ranked as the fifth most popular in Serbia during 2018–2022 [26]. Previous studies encompassing the general canine population in Serbia have shown a relatively low prevalence of *B. gibsoni* among dogs with [3] and without clinical signs [6].

This study aimed to determine the occurrence of *B. gibsoni* and other *Babesia* species in ASTs originating from the Republic of Serbia (including Belgrade) and to identify both animal- and owner-related risk factors associated with *Babesia* spp. infections.

## 2. Materials and Methods

### 2.1. Data Collection and Study Population

This study was approved by the Ethics Committee of the Faculty of Veterinary Medicine, University of Belgrade, Serbia, and permission No 323-07-11564/2022-05/1 was granted by the Ministry of Agriculture, Forestry, and Water Management of the Republic of Serbia. Blood samples were collected from all ASTs and their cross-breeds of both genders and all ages over two years from two veterinary clinics: the Faculty of Veterinary Medicine, University of Belgrade, and the private veterinary clinic PetVetCare whose owners provided informed consent for data usage. All blood samples were included in the study regardless of the reason for the examination, whether for vaccinations, health checks, or the appearance of clinical signs. The only exclusion criterion was that the ASTs had no history of blood transfusion. At the time of each dog’s presentation, owner-related risk factors were recorded, including the administrative district of residence, noting if it is urban or rural, antiparasitic treatment regimen, history of tick exposure, housing conditions (indoor vs. outdoor), and whether the dog resided in a single- or multiple-dog household. Dog-related risk factors included gender, body condition score (BCS) on a scale from 1 to 5, history of vector-borne diseases (VBD), fatigue, fever, and the presence of recent wounds and scars. To examine these risk factors and their relationship with the presence of small *Babesia* species, dogs were divided into two groups: those with clinical signs and those without (clinically healthy). Dogs without significant findings in their history or clinical examination, with a BCS of 3 or higher and without fever, were classified as healthy. In contrast, dogs exhibiting any of the following clinical signs, fever (≥39.3 °C), a BCS of less than 3, and recent wounds were classified as sick. This resulted in creating a database of 101 ASTs with (n = 56) or without health issues (n = 45), residing in rural and urban areas. Blood samples were collected in EDTA tubes and stored at −20 °C until DNA extraction.

### 2.2. Extraction and PCR Detection

DNA was extracted using Thermo Scientific™ GeneJET Whole Blood Genomic DNA Purification Mini Kit, according to the manufacturer’s recommendations from 101 blood samples. To identify *Babesia* parasites, PCR was conducted with specific primers PIRO-A (5′-AATACCCAATCCTGACACAGGG-3′) and PIRO-B (5′-TTAAATACGAAT GCCCCCAAC-3′), targeting a 410 bp fragment of the 18S-rRNA gene of *Babesia* spp. [27]. The PCR reactions were conducted in 25 μL volumes, comprising 1 × PCR buffer (Kapa Biosystems, Wilmington, MA, USA), 1.5 mM MgCl2 (Kapa Biosystems), 100 μM dNTP (Kapa Biosystems), 2 μM of each primer, 0.5 U of Taq polymerase (Kapa Biosystems), and 5 μL of template DNA. Amplification conditions consisted of an initial DNA denaturation step at 95 °C for 3 min, followed by 35 cycles of denaturation at 94 °C for 30 s, annealing at 62 °C for 30 s, extension at 72 °C for 1 min, and a final extension step at 72 °C for 7 min [3]. Amplification products were separated on a 2% agarose gel stained with ethidium bromide and visualized under UV light, with a commercial O’RangeRuler™ 100 bp DNA Ladder (Fermentas, Burlington, ON, USA) serving as a size marker. PCR results showed positive for *Babesia* spp., with a length of approximately 405 bp.

### 2.3. Sanger Sequencing

Specimens that tested positive were further processed for purification using the GeneJET PCR Purification Kit (Thermo Fisher Scientific, Waltham, MA, USA). These samples were then subjected to bidirectional sequencing provided by Macrogen Europe (Amsterdam, The Netherlands). Sequences obtained underwent quality assessments; only those with quality scores exceeding 30 were deemed adequate for additional examination. Initial processing steps involved the excision of primer sequences using the ‘Trim Ends’ tool within the Geneious Prime 2026.0 software (Dotmatics, Boston, MA, USA). A consensus sequence of high quality was established by employing the ‘Generate consensus sequence’ function of the software, with a quality threshold set at 75%. This sequence was subsequently uploaded to the GenBank database at the National Center for Biotechnology Information (NCBI). In this study, the assembled sequences were compared with 49 sequences from various *Babesia* species, including *Babesia gibsoni*, *Babesia canis canis*, and *Babesia vulpes* 18s rRNA, which are available in the NCBI database. Alignment of these sequences was performed using the MAFFT algorithm. Phylogenetic analysis was conducted utilizing the Molecular Evolutionary Genetic Analysis (MEGA X, version 10.2.6) software, employing the Maximum Likelihood method and the Tamura-Nei model, selected via the “Find Best DNA/Protein model” function in MEGA X.

The sequence was submitted to GenBank (NCBI) under accession numbers for *Babesia gibsoni* PP762255–PP762307, and *Babesia vulpes* PP762313–PP762319.

### 2.4. Phylogenetic Analysis

Strains from this study were aligned with 10 strains of *B. vulpes*, and 39 strains of *B. gibsoni* obtained from the NCBI database. Sequences were trimmed to a length of 380 bp further aligned using the MUSCLE algorithm. These aligned sequences were then used for phylogenetic analysis in the Molecular Evolutionary Genetics Analysis (MEGA X) software. The Maximum Likelihood method and Tamura-Nei model, used in the analysis, were determined based on the results from the “Find Best DNA/Protein Models” feature in MEGA X.

### 2.5. Statistical Analysis

The data were analyzed using the IBM SPSS Statistics v20 software package (IBM Corp., Armonk, NY, USA). Pearson’s χ^2^ test and Fisher’s exact test were used for the analysis of categorical variables. Continuous variables were compared using the Mann–Whitney U test, while binary logistic regression was applied to assess risk factors for infection with small *Babesia* species. Additionally, univariable logistic regression analysis of risk factors associated with *Babesia* infection was applied.

## 3. Results

### 3.1. Prevalence and Risk Factors for Small Babesia Species Infection

Initial screening of 101 ASTs showed that 64 were PCR-negative and 37 were PCR-positive for *Babesia* spp. Further, the prevalence for *B. gibsoni* was 35.6% (36/101; 95% CI: 26.3–44.9%), whereas for *B. vulpes* was 1.0% (1/101; 95% CI: 0.2–5.4%).

The proportion of dogs with and without clinical signs was four times lower in PCR-negative than in PCR-positive group (Table 1). Among PCR-positive ASTs, further subdivision by sequencing results and health status revealed that one half of ASTs with clinical signs tested positive for *B. gibsoni* or *B. vulpes*, while a fifth of healthy ASTs tested positive for with *B. gibsoni* (Table 1). The *B. vulpes*-PCR-positive dog, did not have fever, nor wounds, but had BCS = 2.

Analyses of dog-related risk factors indicated that age, body temperature, and weight of ASTs were not risk factors for testing positive for a small *Babesia* species (Table 2).

Furthermore, gender, fatigue and recent wounds were not significant for small *Babesia* infections (Table 3). However, a low BCS and the presence of scars and history of VBD, emerged as significant risk factors (Table 3). History of VBD was reported for 11 dogs; among them one was seropositive to *Anaplasma* spp. and two had confirmed *B. canis* and *B. gibsoni* infection, respectively. For other dogs, owners did not have exact information.

Owner-related risk factors revealed that the type of households and indoor/outdoor housing as well as ectoparasite prevention were not significant risk factors (Table 4). However, tick exposure and life in rural areas were risk factors for testing positive for small *Babesia* species (Table 4).

Table 5 shows the odds ratios and 95% confidence intervals for all significant risk factors. Due to sparse data and category separation, dogs were dichotomized into those with low body condition score (BCS 1–2) and those with non-low BCS (BCS 3–5). The highest odds ratios were observed for living in a rural environment, followed by the presence of scars, tick exposure, a history of vector-borne diseases, and low body condition score (BCS).

### 3.2. Sequencing Results

Out of 37 sequenced samples, 36 were identified as *B. gibsoni* and one as *B. vulpes*, using a BLAST+2.17.0 search provided by NCBI. Regarding *B. gibsoni* the highest percentage of similarity was found with sequences MN134517.1 (India), MH620203.1 (USA), and MN134509.1 (India), exhibiting 100% query coverage and a 100% identity match. Concerning the *B. vulpes* sequence the highest percent identity was recorded with MK585200.1 (Spain), ON968708.1 (Austria), and others with the sequence shared 100% query coverage and a 100% identity match.

### 3.3. Phylogenetic Analysis Results

**Figure 1:**
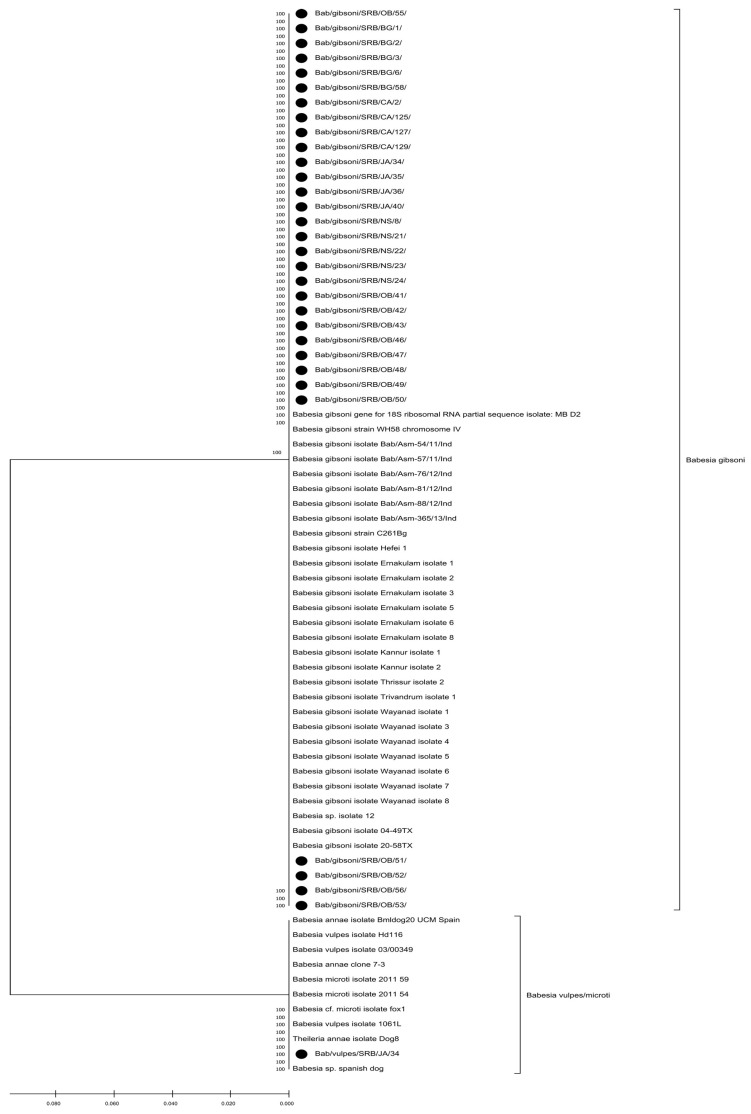
Phylogenetic analysis of *Babesia* spp. based on partial 18S rRNA sequences, with Serbian isolates indicated by black dots.

The phylogenetic tree of the 18S rRNA sequence segment revealed distinct clades corresponding to different *Babesia* spp. Serbian strains of *Babesia* spp. are marked with black dots for easy identification (Figure 1).

A phylogenetic tree was generated to illustrate the relationships between the sequenced *B. gibsoni* (PP762255–PP762285) and *B. vulpes* isolate (PP762316) with representative sequences of other Babesia species retrieved from NCBI. Model selection was performed in MEGA X using the “Find Best DNA/Protein Models” option to determine the most suitable substitution model for the dataset. The tree was constructed with the Maximum Likelihood approach under the Tamura–Nei model, applying 1000 bootstrap replicates to assess branch support. A gamma-distributed rate variation with five discrete categories was used to account for among-site heterogeneity, and invariant sites were included in the model. Branches supported by fewer than 70% of bootstrap replicates were collapsed, and sequences originating from this study were indicated by black dots.

## 4. Discussion

### 4.1. Prevalence of Babesia gibsoni and B. vulpes in ASTs in Serbia

Our results indicate a notably high occurrence of *B. gibsoni* (35.6%) and sporadic infection with *B. vulpes* (1%) in the population of ASTs in Serbia. Similar findings for *B. gibsoni* were shown in ASTs and Pit Bull Terriers in at least two studies: in Hungary, the prevalence was 40.5% [28], and in North America 39% [29]. *Babesia vulpes* was identified in foxes and jackals in Serbia [9], and in 16 asymptomatic stray dogs [8], but this is the first reported case of *B. vulpes* in an owner-dog in a rural region in Serbia. In Spain, a virulent strain of *B. vulpes* (previously referred to as *B. microti*-like) was reported in more than 150 dogs. Another study in Spain, targeting dogs suspected of piroplasmosis found a high prevalence (62.5%) of *B. vulpes* [30]. In Hungary, the prevalence of this small *Babesia* among ASTs was 10.1% [28]. Overall, ASTs in Serbia appear to be a population at substantial risk for *B. gibsoni* infection, with *B. vulpes* emerging as a sporadic but noteworthy finding. This further implies that other similar breeds of dogs should be tested for the presence of small *Babesia* species.

### 4.2. Small Babesia Species Infected ASTs with and Without Clinical Signs

Regarding the distribution of small *Babesia* species in ASTs with and without clinical signs, it is noteworthy that *B. gibsoni*-positive dogs are three times more common in the group of dogs with clinical signs than in the group without clinical signs used to define ASTs in the context of this study. Dogs infected with *B. gibsoni* may exhibit acute or chronic illness or remain asymptomatic [13]. In our study, fever did not emerge as a risk factor, suggesting that acute inflammation is unlikely to be directly associated with small *Babesia* infections. However, acute inflammation may develop in cases where *B. gibsoni* infection acts as a trigger for secondary immune-mediated hemolytic anemia [31]. According to our study, small *Babesia* species are more likely found among ASTs with low BCS, pointing that these infections are primarily chronic, with increased catabolism and muscle tissue loss. Our study revealed nine dogs with asymptomatic *B. gibsoni* infection. Namely, natural or experimental infections with *B. gibsoni* [32,33] or *B. vulpes* may be asymptomatic [13,34], possibly being dependent on the pathogenicity of small *Babesia* strain, the immune status and age of the host [35].

It is interesting to note that at the presentation, the *B. vulpes* infected dog did not have a fever, but had BCS = 2, did not have antiparasitic prophylaxis, was exposed to ticks, had scars, and lived outdoors in a multi-dog household. From these observations, it could be concluded that risk factors for *B. vulpes* infection were in line with those for *B. gibsoni* infection. In northwestern Spain, *B. vulpes* infection was linked to acute kidney failure and high mortality [30], whereas the cases reported in Serbia—including the one in the present study—were asymptomatic or, as suggested here, possibly chronic, implying geographical difference in the strains of this small *Babesia*.

### 4.3. Transmission Routes of Small Babesia Species: Dog Bites vs. Tick Exposure

The presence of scars was identified as a significant dog-related risk factor, indicating that previous dog bites played an important role, as reported earlier [14,15]. Currently, in Europe and North America, ASTs used for fighting have been shown to exhibit a high prevalence of *B. gibsoni* infection [17,18,28]. Also, it is reported that ASTs have a high frequency of aggression toward other dogs [36], explaining the presence of scars in our study as a consequence of dog-to-dog bites. However, direct evidence that dog bites are a way of small *Babesia* transmission is lacking. Conducting an experimental study to unequivocally confirm this hypothesis would pose significant ethical challenges, which explains the lack of definitive proof. Having that in mind, our findings offer a broader perspective, showing that recent wounds do not pose a significant risk, whereas the presence of scars is a risk factor for small *Babesia* infections. Explanation may be that detectable parasitemia does not establish immediately after a bite, and that exceeds the time required for wound healing. The time required for parasitemia to reach levels detectable by molecular methods, following natural infection—whether via a dog bite or a tick bite—remains unknown. Nevertheless, the history of VBD was also a risk factor for *B. gibsoni* infection, meaning that some dogs were constantly exposed to ticks. The anamnesis revealed that owners were poorly informed about the etiological agents that caused infections. Nevertheless, one owner documented that his dog was exposed to *Anaplasma* spp., and two owners reported infections with *B. canis* and *B. gibsoni*. As exposure to ticks and living in a rural area are risk factors, it is possible that ticks present in Serbia could transmit *B. gibsoni*. Presently, it is postulated that *R. sanguineus* could be the relevant vector for *B. gibsoni* transmission [17]. This, and similar studies [4] underscore the necessity for broader screening of ticks to establish a true vector for *B. gibsoni* in Serbia and Europe. Two-thirds of owners of *Babesia*-positive ASTs reported using antiparasitic prophylaxis, suggesting that some owners who claimed to use antiparasitic prophylaxis may not have applied it correctly. Notably, none of the ASTs—whether clinically healthy or with clinical signs—tested positive for *B. canis*, a widespread vector-borne pathogen in Serbia, known to cause acute [3] or asymptomatic infections [37]. This indicates that ASTs were not exposed to *Dermacentor reticulatus* infected with *B. canis*. However, given that living in rural areas emerged as the most significant risk factor, ticks should be thoroughly explored as vectors important for transmission.

### 4.4. Possible Vertical Transmission of Small Babesia Species and Other Risk Factors

Our study revealed nine *B. gibsoni* infected ASTs that were clinically healthy and without scars. This is an important finding as it implies that a substantial proportion of infections within a specific breed, such as ASTs and related breeds, may persist through tick bites or vertical transmission. We hypothesize that this last possibility at least partially explains the high prevalence of *B. gibsoni* in our study. This is further supported by our recent work that found *B. gibsoni* in the semen of infected ASTs [38] and a report showing its transplacental transmission [21]. Vertical transmission may contribute to the maintenance and spread of small *Babesia* species in wild canids.

The lack of a clear association between age and small *Babesia* infections remains puzzling. Given that *B. gibsoni* typically causes chronic infection, a higher prevalence in older dogs would be expected. An alternative explanation is that infected dogs may die prematurely due to inadequate treatment or the limited efficacy of available chemotherapeutics. Currently, two commonly used treatment protocols include a 10-day course of atovaquone combined with azithromycin [39] and a 21-day combination of clindamycin, metronidazole, and doxycycline [40]. However, both regimens have limited success in fully eliminating the parasite, resulting in many dogs remaining chronic carriers and potential reservoirs, which contributes to further dissemination and increases the likelihood of relapse. Other dog-related factors, such as weight, sex, and fatigue, do not appear to substantially increase the suspicion of small *Babesia* infection in ASTs. Interestingly, although most ASTs are kept in multi-dog households and housed outdoors, neither factor emerged as a significant risk in this study.

### 4.5. Genealogy of Small Babesia Species

The sequenced *B. gibsoni* strains were identical to each other, suggesting that the 18S region sequenced with PIRO A and B primers is highly conserved and unsuitable for further classification of *B. gibsoni* strains. According to the recent classification proposed by Baneth et al. [41], the other small *Babesia* found in our study can be classified as *Babesia vulpes*. The sequenced strain of *B. vulpes* was compared to previously published sequences from Serbia (MH699381–MH699396), and in the overlapping regions, no differences were found. Further classification is beyond the scope of this study and will be addressed in future research.

### 4.6. Limitations of the Study

Concerning the prevalence and the true number of infected dogs, two additional analyses could improve the accuracy of the findings: 1. Multiple PCR Testing: Repeated PCR testing could identify dogs that did not have detectable parasitemia at the time of sampling. For instance, dogs infected with *B. gibsoni* may only intermittently test PCR-positive [42]. The same intermittent detection might apply to *Babesia vulpes* infections; 2. Molecular and serological data combination: A more accurate estimation of the number of dogs infected with small *Babesia* species could be achieved by integrating molecular data with seroprevalence studies. Regarding the usefulness of molecular data, additional analyses are suggested: Alternative primers: Utilizing primers designed to better assess the genetic diversity of small *Babesia* species and classify them into specific clades [43] would highlight the possible routes of infection (bite wounds vs. vertical transmission) and would underline the potential presence of zoonotic clades. Finally, concerning the evaluation of risk factors, a more detailed questionnaire is recommended. This should include data on the genealogical trees of ASTs to explore potential hereditary or lineage-related risk factors.

## 5. Conclusions

This study holds significant genetic and epizootiological value. It revealed a high prevalence of *B. gibsoni* and the first detection of *B. vulpes* in Serbia’s AST population. Key risk factors include dog bites and tick exposure; however, the role of vertical transmission warrants careful evaluation. Regular testing of ASTs and related breeds, even asymptomatic dogs, during annual check-ups, as well as further identification of risk factors, is essential to prevent and control the disease.

## Figures and Tables

**Table 1:** Prevalence of *Babesia gibsoni* and *B. vulpes* in PCR-positive American Stafford Terriers (ASTs) with and without clinical signs. Percentages in the PCR-negative and PCR-positive columns are calculated by taking the absolute number of PCR-negative and PCR-positive ASTs, respectively, as 100%. *p*-value shows the difference between the number of *B. gibsoni* and *B. vulpes* in healthy and sick dogs.

	Negative n = 64	Positive n = 37	*p*-Value
With/without clinical signs	0.7	3.1	Fisher’s Exact *p* = 0.003
With clinical signs	28 (43.8%)	28 (75.6%)	
*B. gibsoni*	/	27 (72.9%)	
*B. vulpes*	/	1 (2.7%)	
Without clinical signs	36 (56.2%)	9 (24.4%)	
*B. gibsoni*	/	9 (24.4%)	
*B. vulpes*	/	0	

**Table 2:** Dog-related risk factors for small *Babesia* PCR-positive findings. The results are shown as median (minimum–maximum). Mann–Whitney U test, *p* < 0.05 is considered significant. The absolute number of dogs with and without clinical signs is given in superscript.

	PCR-Negative n = 64	PCR-Positive n = 37	*p*-Value
	With clinical signs		
Body temperature (°C)	39 (37–41)^28^	38 (37–40)^28^	*p* = 0.058
Weight (kg)	18 (10–33)^28^	20 (15–28)^28^	*p* = 0.097
Age (years)	4 (2–9)^28^	4 (2–9)^28^	*p* = 0.555
	Without clinical signs		
Body temperature (°C)	38 (37–38)^36^	38 (37–38)^9^	*p* = 0.686
Weight (kg)	21.5 (15–41)^36^	18 (15–30)^9^	*p* = 0.267
Age (years)	4 (2–9)^36^	3 (2–8)^9^	*p* = 0.393

**Table 3:** Dog-related risk factors for small *Babesia* PCR-positive findings. Pearson’s Chi-square test, Fisher’s exact test; *p* < 0.05 is considered significant.

	PCR-Negative n = 64	PCR-Positive n = 37	*p*-Value
Gender			*p* = 0.216
Male	45.3%	59.5%	
Female	54.7%	40.5%	
Fatigue			*p* = 0.059
Yes	1.6%	10.8%	
No	98.4%	88.2%	
Recent wounds			*p* = 0.766
Yes	12.5%	16.2%	
No	87.5%	83.8%	
Scars			*p* = 0.001
Yes	60.9%	91.9%	
No	39.1%	8.1%	
BCS			*p* < 0.001
1	1.6%	13.5%	
2	9.5%	35.1%	
3	31.7%	21.6%	
4	36.5%	21.6%	
5	20.6%	8.1%	
History of VBD			*p* = 0.035
Yes	4.7%	18.9%	
No	95.3%	81.1%	

BCS: Body condition score; VBD: Vector-borne disease.

**Table 4:** Owner-related risk factors for small *Babesia* PCR-positive findings. Percentages in the PCR-negative and PCR-positive columns are calculated by taking the absolute number of PCR-negative and PCR-positive ASTs, respectively, as 100%. Pearson’s Chi-square test, Fisher’s exact test, *p* < 0.05 is considered significant.

	PCR-Negative n = 64	PCR-Positive n = 37	*p*-Value
Households			*p* = 0.531
Single-dog	3.1%	0.0%	
Multi-dog	96.9%	100.0%	
Housing			*p* = 0.205
Indoor	29.7%	16.2%	
Outdoor	70.3%	83.8%	
Antiparasitics			*p* = 0.105
Yes	62.5%	67.6%	
No	37.4%	32.4%	
Ticks’ exposure			*p* = 0.004
Yes	3.1%	21.6%	
No	96.9%	78.4%	
District			*p* < 0.001
Urban	76.5%	23.5%	
Rural	36.4%	63.6%	

**Table 5:** Univariable logistic regression analysis of risk factors associated with small *Babesia* species infection.

	Odds Ratio	95% Confidence Intervals	*p*-Value
Rural environment	5.68	2.30–14.08	<0.001
Presence of scars	4.00	1.74–9.19	0.001
Ticks exposure	3.53	0.90–13.86	0.071
Previous vector-borne disease	2.99	0.88–10.18	0.080
Low body condition score	2.91	1.18–7.19	0.021

## Data Availability

The original contributions presented in this study are included in the article. Further inquiries can be directed to the corresponding author.
